# Gastric Perforation Due to Sarcina ventriculi: A Histologically Confirmed Case

**DOI:** 10.7759/cureus.32609

**Published:** 2022-12-16

**Authors:** Linda C Klumpp, Brenton Sinopoli, Bailey L Alkhatib, Joseph Horvath

**Affiliations:** 1 Infectious Diseases, Prisma Health Richland/University of South Carolina, Columbia, USA; 2 Internal Medicine, Prisma Health Richland/University of South Carolina, Columbia, USA; 3 Internal Medicine, University of South Carolina School of Medicine, Columbia, USA

**Keywords:** delayed gastric emptying, gram negative coccus, ulcer, pathology, histology, gastritis, sarcina ventriculi, gastric perforation

## Abstract

We present a case of a rare cause of gastric perforation and pneumoperitoneum, associated with *Sarcina ventriculi*. An 88-year-old male presented to the emergency room with significant abdominal pain as his chief complaint. Abdominal radiograph showed extensive free intraperitoneal gas under the diaphragms. Computed tomography (CT) of the abdomen and pelvis showed pneumatosis, portal venous gas, and extensive free intraperitoneal gas with free fluid. Immediate surgical intervention ensued. The gastric biopsies obtained proved valuable for confirming the diagnosis of *S. ventriculi. *In conjunction with surgery, the patient received a course of antibiotics for a cure.

## Introduction

*Sarcina ventriculi* is a gram-positive, non-motile, anaerobic coccus that can flourish in an acidic environment [1]. The bacterium was first described in 1842 in an individual suffering from an affliction of the digestive tract [1]. Microscopy of gastric mucosa showed small packets arranged in tetrads or octets, hence the name “Sarcina”, Latin for package. This histologic finding as well as its ability to form spores can differentiate it from other bacteria [1,2]. The natural habitat of *Sarcina *species is the soil.

Despite being described on microscopy in 1842, it was not until 1911 that the bacteria were isolated in pure culture under strict anaerobic technique [1,3]. Histologically the bacteria are located on the gastric mucosal surfaces and are non-pathogenic. Based on case reviews, the most associated clinical and histological signs are abdominal pain, food bezoar, and gastric ulcer [1]. Growth of* S. ventriculi *may occur in the human stomach because of delayed gastric emptying from pathologic conditions such as diabetic gastroparesis, gastric surgeries, scarring, pyloric stenosis, or an obstructing mass [1,2]. Acute and or chronic gastritis is the most common finding in histology. There have been 66 reported cases of *S. ventriculi *implicated in human disease; our case may be the 67th case, and the third documented case associated with gastric perforation [1]. Our case is unique as our patient did not have a history of peptic ulcer disease or risk factors for delayed gastric emptying.

## Case presentation

An 88-year-old male presented to the emergency room with complaints of moderate abdominal pain, nausea, and vomiting. On initial presentation, he was noted to have tachycardia with a heart rate of 121 beats per minute, and a blood pressure of 165/91 mmHg. 

Medical history was significant for hypertension, grade I diastolic dysfunction, history of prior pulmonary embolism, and deep vein thrombosis. The patient had completed a treatment course with anticoagulation. Surgical history was significant for hernia repair. The patient appeared malnourished and there was a concern for neglect.

**Figure 1:**
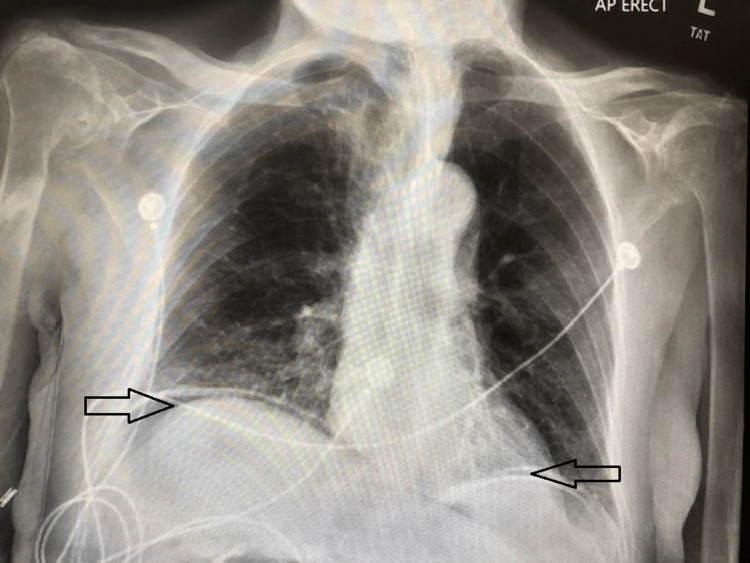
Bilateral free air under the diaphragms (arrows)

**Figure 2:**
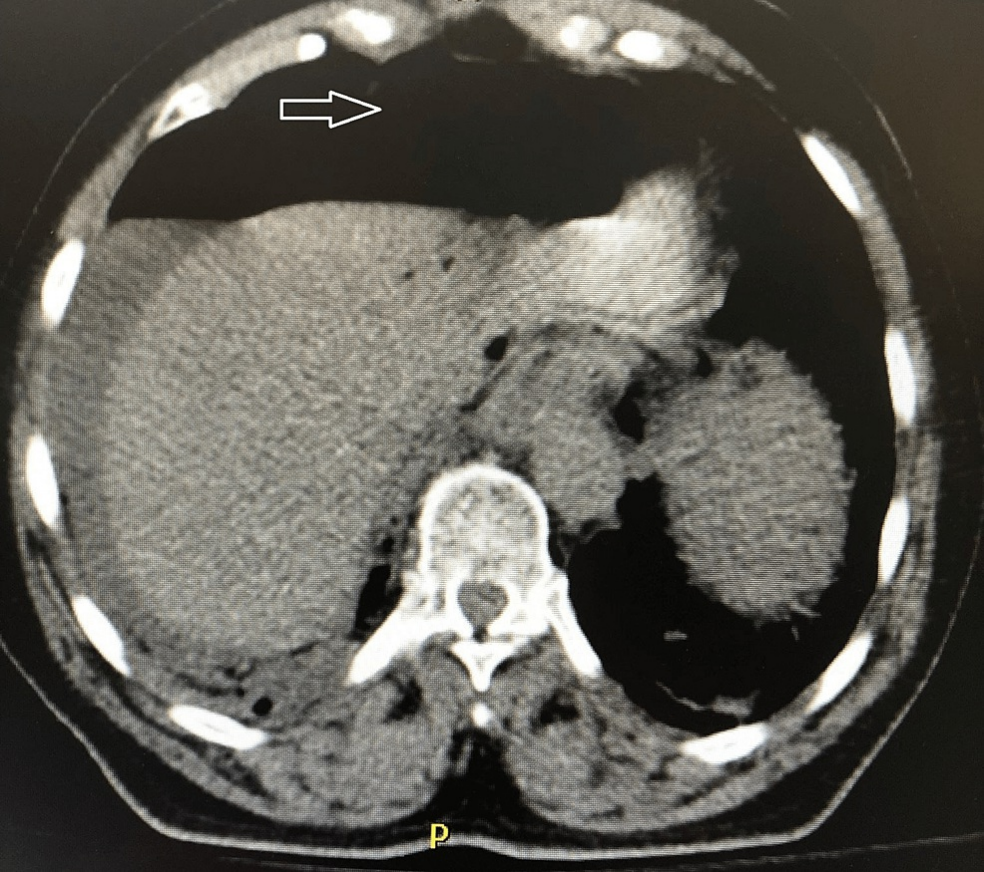
CT showing free air (arrow)

On physical exam, the patient was noted to have severe bilateral upper quadrant pain, a ventral hernia, and an irreducible inguinal hernia. Laboratory findings were consistent with metabolic acidosis; the anion gap was 22. There was no evidence of leukocytosis. Of note, the patient was hyperglycemic with a blood glucose of 193, but he did not have a documented history of diabetes mellitus. Imaging studies proved valuable as the patient needed surgical intervention. Chest radiograph showed bilateral free air under the diaphragms (Figure 1). CT scan showed pneumatosis, portal venous gas, and extensive free intraperitoneal gas with free fluid (Figure 2).

General surgery was immediately notified, and the patient underwent exploratory laparotomy with findings of a 1.00 cm anterior wall gastric perforation without an ulcer, with a ventral hernia and recurrent incarcerated left inguinal hernia. On initial entry into the abdominal cavity, succus was noted. There was a noted ventral hernia with an incarcerated omentum - this was removed. The incarcerated inguinal hernia was noted to be indirect and contained colon. The hernia sac was ligated and easily reduced. The gastric perforation was then repaired with silk sutures and reinforced with an omental plug (Graham patch). Gastric biopsies were obtained.

**Figure 3:**
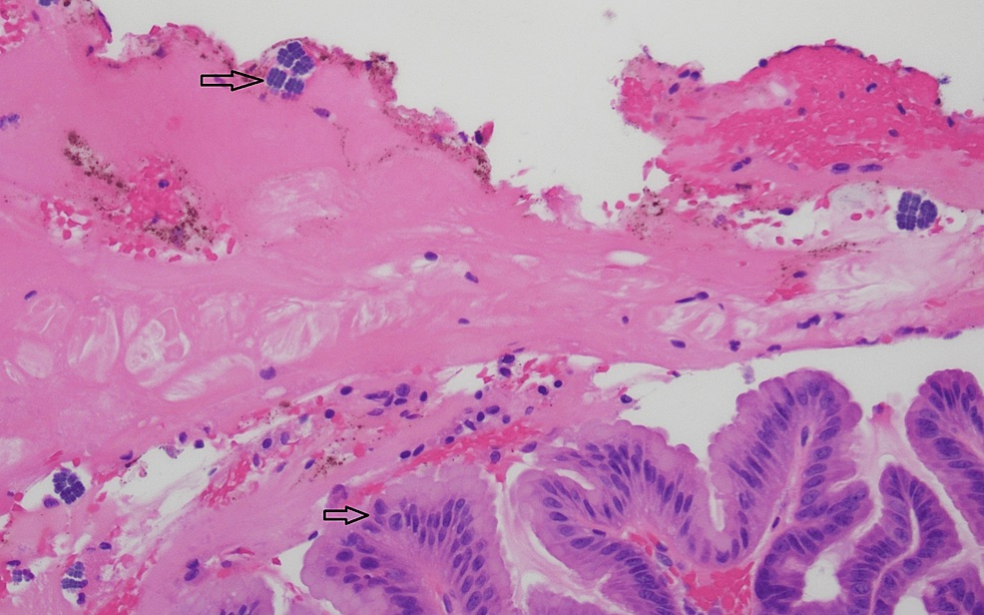
Hematoxylin and eosin stain of gastric epithelium under microscopy at 20X The top arrow indicates the cuboid and tetrad packets of *Sarcina ventriculi*; the bottom arrow points to gastric epithelium and pits.

**Figure 4:**
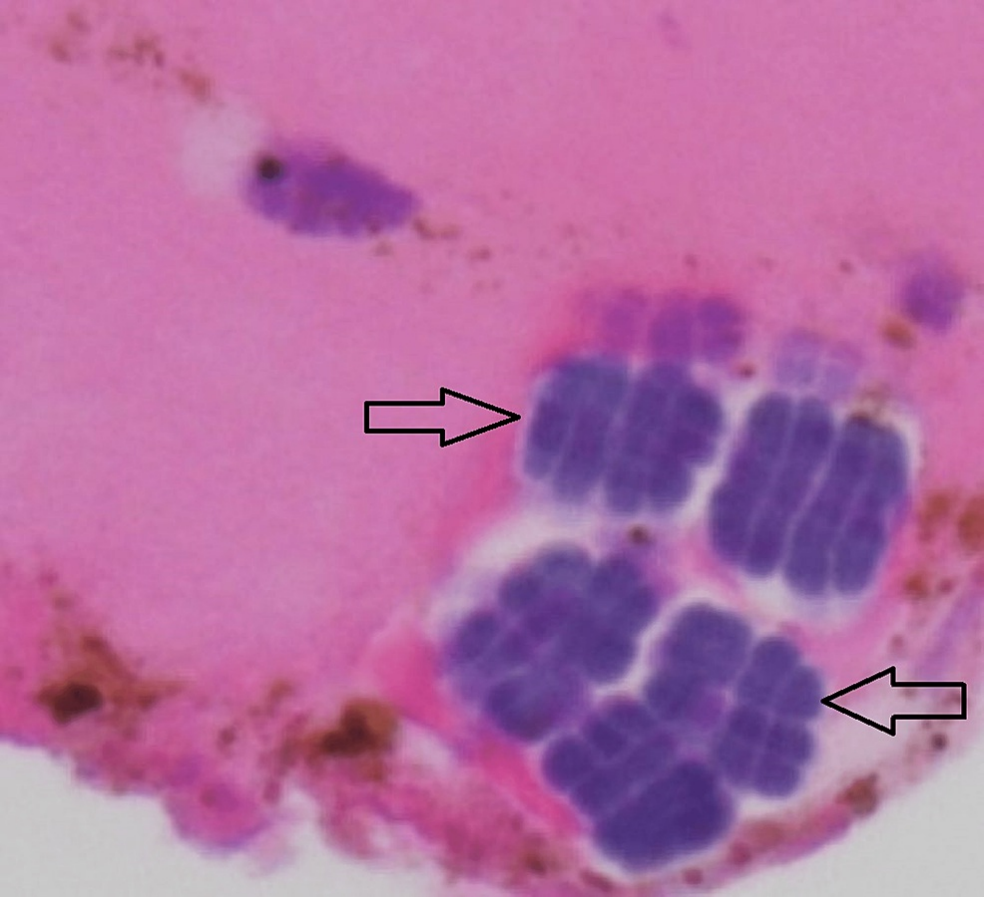
Hematoxylin and eosin stain at 40X showing S. ventriculi cuboids and tetrad packets (arrows)

Pathology findings from the partial gastrectomy showed fibroadipose soft tissue and gastric epithelium with acute and chronic inflammation with abscess formation and bacterial forms, most compatible with *Sarcina ventriculi* (Figures 3, 4). The organism was identified by light microscopy with characteristic findings of basophilic staining with hematoxylin and eosin, cuboid shape, and tetrad packets. Organisms were located in the mucosal layers.

After identification of the bacteria, the patient was treated with a 14-day course of oral ciprofloxacin 500 mg two times per day and oral metronidazole 500 mg three times a day. The patient made a full recovery and was discharged home.

## Discussion

*Sarcina ventriculi *is a rare cause of gastric perforation and its pathogenic role is still not fully understood. The bacteria are proven gram-positive, non-motile, anaerobic coccus with an exclusive carbohydrate fermentative metabolism [1]. The products of fermentation are ethanol, acetaldehyde, carbon dioxide, and hydrogen [1]. *S. ventriculi *exists naturally in the soil and has been associated with gastric dilation and death in livestock. In their natural habitat, the bacteria form spores and can survive for years at an acidic pH [1,4]. The bacteria have been isolated in the feces of healthy humans, particularly in vegetarians. It is often discovered as an incidental finding on microscopy using hematoxylin and eosin staining. It tends to be located near the gastric mucosa and is not invasive. The bacteria have only been isolated under strict anaerobic conditions, which makes obtaining culture difficult.

In our literature review, we found two documented cases that had severe complications of emphysematous gastritis and peritonitis with underlying ulcers; researchers surmised that a preexisting mucosal defect may provide a nidus for emphysematous gastritis as opposed to direct invasion [1]. Also, it is unclear if local accumulation of acetaldehyde and ethanol formed from carbohydrate fermentation could induce mucosal injury similar to alcohol ingestion [4]. The bacteria can be differentiated from other species that may have a tetrad or package-forming arrangement, in particular, micrococcus species, which is aerobic, catalase-positive, and does not form spores.

As previously mentioned, the most common presenting symptoms of infection have been abdominal pain, nausea, and vomiting. These symptoms have been primarily associated with gastric delayed emptying. Gastric ulcer and gastritis are the most common endoscopic findings. It is unclear if there is a relationship between *S. ventriculi *and *Helicobacter pylori*. There has been one documented case of *H. pylori* and *S. ventriculi* in the same specimen, and one documented case in which *S. ventriculi* was present after the eradication of* H. pylori*. 

Diagnosis can be confirmed by culture, histology, or confirmed with 16S ribosomal RNA.

Treatments have been based on animal studies, which proved fluoroquinolones, macrolides, penicillin, and tetracyclines are most effective for symptom improvement and eradication. Proton pump inhibitors may also contribute to symptom relief.

## Conclusions

In patients with known gastric ulcers, gastric surgery or conditions related to delayed gastric emptying, suspicion for *Sarcina ventriculi* should remain high. Most cases are biopsy proven as opposed to culture proven. Suspicion should be further escalated in the event of a life-threatening gastric perforation and emphysematous gastritis. Gastric perforation may occur in the absence of an evident ulcer, as in our case. *Sarcina ventriculi* can be treated with fluoroquinolones and metronidazole for eradication. As cases of *Sarcina ventriculi *are increasing, more studies are needed to further investigate whether the bacteria are truly innocent bystanders or if an underlying pathological process exists.
